# Determination of the Strain Influence on the InAs/InAsSb Type-II Superlattice Effective Masses

**DOI:** 10.3390/s22218243

**Published:** 2022-10-27

**Authors:** Tetiana Manyk, Jarosław Rutkowski, Małgorzata Kopytko, Piotr Martyniuk

**Affiliations:** Institute of Applied Physics, Military University of Technology, 2 Kaliskiego Str., 00-908 Warsaw, Poland

**Keywords:** type-II superlattice, k·p method, infrared detectors, effective masses

## Abstract

A^3^B^5^ materials used for the superlattice (SL) fabrication have properties that enable the design of devices optimized for infrared (IR) detection. These devices are used in the military, industry, medicine and in other areas of science and technology. The paper presents the theoretical assessment and analysis of the InAs/InAs_1−*x*_Sb*_x_* type-II superlattice (T2SL) (grown on GaSb buffer layer) strain impact on the bandgap energy and on the effective masses of electrons and holes at 150 K. The theoretical research was carried out with the use of the commercial program SimuApsys (*Crosslight*). The k·p method was adopted in T2SL modeling. Luttinger coefficients (*γ*_1_, *γ*_2_ and *γ*_3_) were assessed assuming the Kane coefficient *F* = 0. The bandgap energy of ternary materials (InAs_*x*_Sb_1−*x*_) was determined assuming that the bowing parameter (*b*_g_) for the above-mentioned temperature is *b*_g_ = 750 meV. The cutoff wavelength values were estimated based on the theoretically determined absorption coefficients (from approximation the quadratic absorption coefficient). The bandgap energy was calculated according to the following formula: *E*_g_ = 1.24/*λ*_cutoff_. The theoretical simulations allowed us to conclude that the strain in T2SL causes the *E*_g_ shift, which also has an impact on the effective masses *m*_e_ and *m*_h_, playing an important role for the device’s optical and electrical performance. The T2SLs-simulated results at 150 K are comparable to those measured experimentally.

## 1. Introduction

The InAs/InAs_1−*x*_Sb*_x_* type-II superlattices (T2SLs) lattice matched to GaSb have recently been proposed as a HgCdTe alternative for medium to long infrared photodetectors [1,2,3,4] due to the stronger immunity to tunneling and reduced Auger generation. Lattice constants of the T2SL materials are around 6.1 Å [5], and hence can be grown on a GaSb substrate. The InAs/InAs_1−*x*_Sb*_x_* T2SLs are promising materials for infrared applications [6,7]. The T2SLs InAs/InAs_1−*x*_Sb*_x_* bandgap energy mostly depends on the SL layers thickness and on the Sb molar composition. In addition, the T2SLs bandgap energy is closely related to the strain within structure. Effective masses of electrons and holes are important material parameters affecting most carrier transport properties. In the papers [8,9], the studies of electrons and holes effective masses were carried out for T2SL with the Sb molar composition *x* = 0.4 and *x* = 0.5 (for the LWIR range). The smaller hole and electron effective masses can lead to reduced scattering, what explains the high quantum efficiency that has been observed for InAs/InAs_1−*x*_Sb*_x_* T2SL detectors [8,9]. The research on the InAs/InAs_1−*x*_Sb*_x_* T2SLs effective masses for the MWIR range is quite important, because detectors optimized for this cutoff wavelength are widely used in safety industry, medicine and for a wide range of applications in the fields of science and technology. In this paper, we present the results of simulations performed for InAs/InAs_1−*x*_Sb*_x_* T2SLs by the k·p method [2,10]. We considered T2SLs grown on GaSb substrate and determined the absorption edge and band structure at the temperature *T* = 150 K. We focus on the effective masses T2SL as a function of the molar composition and period thickness of the superlattice. We present our results in order to show the effect of the strain on the electron and heavy-hole effective masses in T2SL.

## 2. Materials and Methods

**Figure 1:**
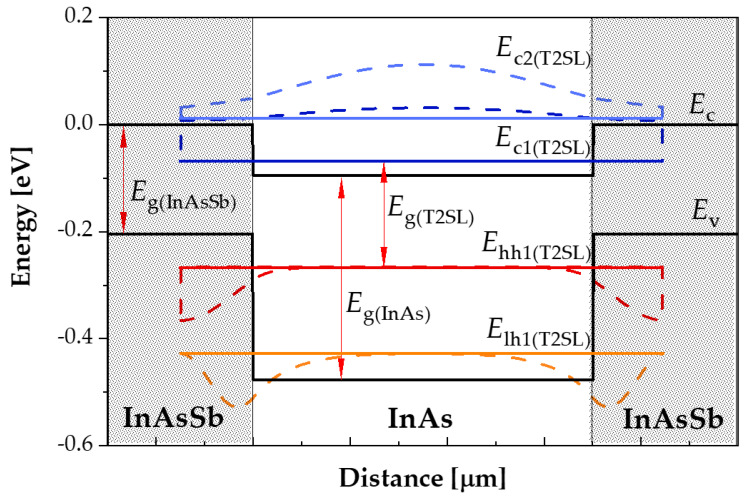
Plot of the T2SL energy bands. (*E*_c_—conduction band, *E*_v_—valence band, *E*_g_—bandgap energy, *E*_c1,c2_—electron band, *E*_hh1_—heavy hole band, and *E*_lh1_—light hole band).

The T2SL made of InAs and InAs_1−*x*_Sb*_x_* exhibits a strain. For their bandgap energy, they have applications found in medium-wave (MWIR) and long-wave (LWIR) IR detector capabilities (the bandgap energy that can vary from 3 µm to 30 µm by changing the thicknesses of the components and the Sb molar composition). The InAs/InAs_1−*x*_Sb*_x_* T2SL consists of a large number of thin periodically stacked InAs and InAs_1−*x*_Sb*_x_* layers. The k·p method was used in the theoretical modeling the T2SL. This method makes it possible to obtain the band structure of the superlattice as well as to estimate the absorption coefficient. The idea of method k·p is to solve the Schrödinger equation [11,12]. InAs/InAs_1−*x*_Sb*_x_* T2SL was simulated by the commercial software SimuApsys (*Crosslight*). Figure 1 shows schematic energy band of the T2SL without considering the strain in InAs and InAs_1−*x*_Sb*_x_*. The electrons in InAs/InAs_1−*x*_Sb*_x_* T2SLs are confined in InAs, while holes are confined in InAs_1−*x*_Sb*_x_*. The bandgap energy of T2SLs is defined as the energy gap between the bottom of the lowest electron mini-band (*E*_c1_) and the top of the highest hole mini-band (*E*_hh1_) (see Figure 1). 

The black line represents the InAs_1−*x*_Sb*_x_* and InAs conduction and valence bands, while blue and red line represents the electron and hole mini-bands (heavy—hh1 and light—lh1) in InAs/InAs_1−*x*_Sb*_x_* T2SL. The blue and red dashed line represents the electron and hole density, respectively. Electrons for InAs/InAs_1−*x*_Sb*_x_* T2SL are confined in InAs, while holes in InAs_1−*x*_Sb*_x_*. The band structure calculation is an important step of detector modeling [13]. The T2SLs bandgap energy is defined as the energy between the bottom of the lowest electron mini-band and the top of the highest hole mini-band (see Figure 1).

**Figure 2:**
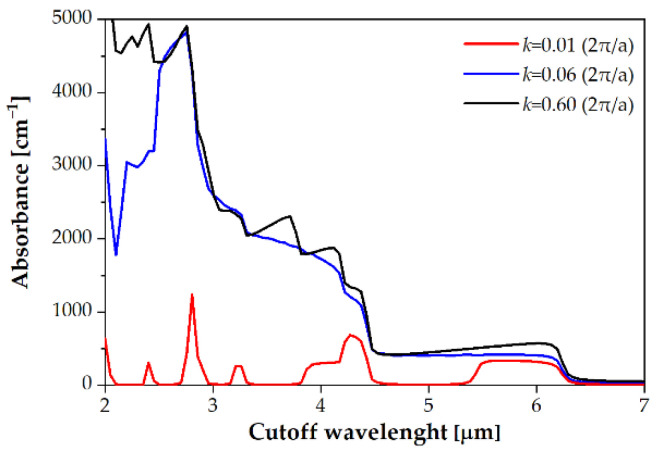
Theoretically calculated absorption coefficients versus wavelength for three wavevector ranges: *k* = 0.01 (2π/*a*), *k* = 0.06 (2π/*a*) and *k* = 0.6 (2π/*a*).

First, an analysis of the absorption coefficient, *α*, versus cutoff wavelength was performed to evaluate the bandgap energy (*E*_g_ = 1.24/*λ*_cutoff_). Figure 2 shows the example of theoretically calculated absorption coefficients for 150 K (Sb molar composition *x* = 0.38) for three wavevector range: *k* = 0.01 (2π/*a*), *k* = 0.06 (2π/*a*) and *k* = 0.6 (2π/*a*). Figure 2 shows that the higher absorption coefficient corresponds to higher wavevector, but the position of cutoff wavelength does not change. In the modeling, data from Table 1 were used. The overlap between the conduction band (*E*_c_) of InAs and the valence band (*E*_v_) of InAs_1−*x*_Sb*_x_* in our simulations was accepted in the 90–140 meV range.

The bandgap energy was extracted from the quadratic absorption coefficient approximation [13]. The energy gap determined in this way is consistent with the value calculated from the energy bands (see Figure 1). The absorption coefficient assumes 2000–7000 cm^−1^ within MWIR range. These simulations provide a good estimate of the bandgap energy in InAs/InAs_1−*x*_Sb*_x_* T2SLs system. The parameters of InAs, InAs_1−*x*_Sb*_x_* and GaSb layers used in InAs/InAs_1−*x*_Sb*_x_* T2SL simulation procedure were taken from papers [5,13,14,15,16,17,18]. The bandgap energy versus temperature is expressed by the Varshni equation [7]. All parameters of the ternary materials InAs_1−*x*_Sb*_x_* used for the theoretical simulation were estimated based on the bulk parameters for binary compounds (InAs and InSb) [13].

The bowing coefficient of the InAs_1−*x*_Sb*_x_* bandgap energy assumes *b*_g_ = 750 meV [5,7]. The Luttinger parameters (*γ*_1_, *γ*_2_, *γ*_3_) were estimated based on the respective InAs_1−*x*_Sb*_x_* effective masses (*m*_lh_, *m*_hh_, *m*_so_) [11]. An important input parameter is the thickness of the SL components (*L*_InAs_ and *L*_InAsSb_) balancing the strain. The InAs_1−*x*_Sb*_x_* layer thickness (*L*_InAsSb_) versus the Sb molar composition (*x*) and T2SL period (*L*_T2SL_) can be calculated by setting the average lattice parameter of one period weighted with the layer thickness equal to the GaSb lattice parameter [5,19]. We considered the symmetrical case where the Kane parameter is equal to zero (*F* = 0). The input parameter equation for the InAsSb ternary material is defined as:

$$E_{g\_\mathrm{InAsSb}}=\left(1-x\right)E_{g\_\mathrm{InAs}}+xE_{g\_\mathrm{InSb}}-b_{g}x\left(1-x\right)$$

Table 1 shows the main input data for SL modeling (for example *x* = 0.30). The lattice constant of the ternary materials InAs_1−*x*_Sb*_x_* was determined using Vegard’s rule.

The thickness (period) InAs/InAs_1−*x*_Sb*_x_* T2SLs is the sum of the thickness InAs and InAs_1−*x*_Sb*_x_*: *L*_T2SL_ = *L*_InAs_ + *L*_InAsSb_. In this paper, we considered three examples T2SLs:

1. Strain balanced (unstrain)—where the thicknesses of individual SL layers meet the condition according to the formula:

(1) $$L_{\mathrm{InAsSb}}=\left(\frac{L_{T2\mathrm{SL}}}{x}\right)\times \left(\frac{a_{\mathrm{GaSb}}-a_{\mathrm{InAs}}}{a_{\mathrm{InSb}}-a_{\mathrm{InAs}}}\right)$$

where *a*_GaSb_, *a*_InAs_ and *a*_InSb_ are the lattice constants for the respective binary materials, *x*– is the Sb molar composition for InAs_1−*x*_Sb*_x_*.

2. Aligned (strain)—thicknesses of InAs and InAs_1−*x*_Sb*_x_* layers are the same: *L*_InAs_ = *L*_InAsSb_.

3. Inverted versus balanced (strain)—thicknesses of individual SL layers are assessed according to the Formula (1) and reversed with values

(2) $$L_{\mathrm{InAs}}=\left(\frac{L_{T2\mathrm{SL}}}{x}\right)\times \left(\frac{a_{\mathrm{GaSb}}-a_{\mathrm{InAs}}}{a_{\mathrm{InSb}}-a_{\mathrm{InAs}}}\right)$$

**Figure 3:**
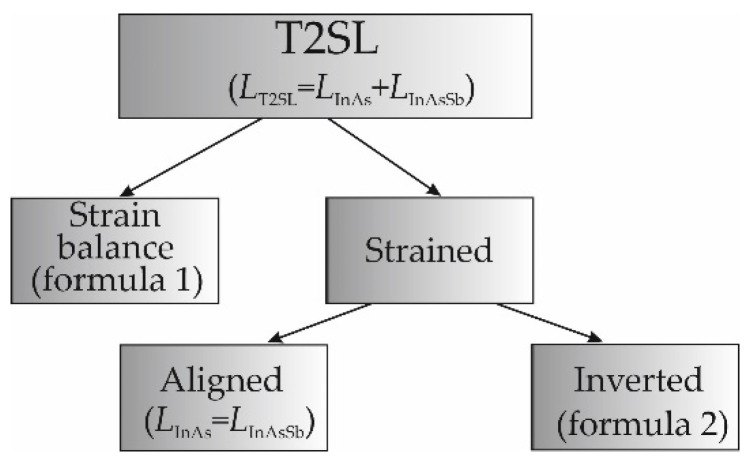
Scheme for determining the thickness of the tested T2SLs.

The estimation of the thickness of individual layers (InAs and InAs_1−*x*_Sb*_x_*) of the tested SL is shown in Figure 3.

## 3. Results and Discussion

**Figure 4:**
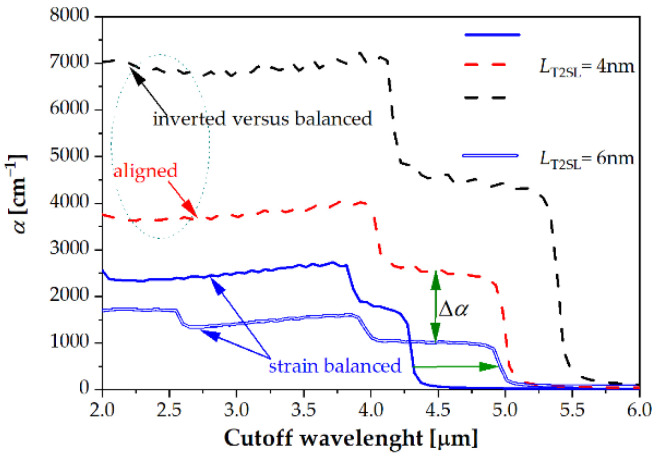
Theoretical simulation of the absorption coefficients for the strain balanced and strained SL at *T* = 150 K for the layer thickness *L*_T2SL_ = 4 nm (*x* = 0.30) and *L*_T2SL_ = 6 nm (*x* = 0.30).

We presented the electrons and holes effective masses, bandgap energy and absorption calculated by the k·p method [11,12]. Figure 4 shows the calculated absorption coefficients for strain-balanced and strained SL. The period of the investigated T2SL is 4 nm. Note that the blue curve corresponds to the strain balanced T2SL structures on GaSb. The red and black curves correspond to the strained structure. These two structures differ in the thickness of the InAs_1−*x*_Sb*_x_* layers (with a constant SL period). The red curve corresponds to SL, which has the thickness of InAs equal to the thickness of InAs_1−*x*_Sb*_x_*. The black curve corresponds to T2SL with the inverted thickness of InAs and InAs_1−*x*_Sb*_x_* layers.

When the strain increases, the absorption coefficient rises, and the values of cutoff wavelength (*λ*_cutoff_) shifts towards a longer wavelength. The higher T2SL strain, the smaller the SL period thickness through which the required bandgap energy can be reached. For example, the blue double line represents the balanced SL for the layer thickness of 6 nm with the same cutoff wavelength of 5 µm as the unbalanced red one with *L* = 4 nm. However, as the strain in ternary InAs_1−*x*_Sb*_x_* increases, the InAs/InAs_1−*x*_Sb*_x_* T2SL absorption coefficient for the same energy gap (Δ*α* on Figure 4) increases, which in the end may turn out to be an advantage of unbalanced versus balanced.

**Figure 5:**
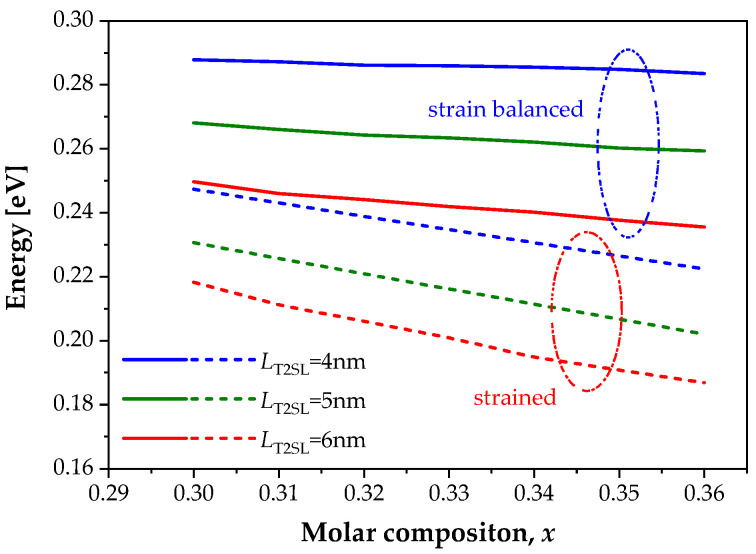
Theoretical simulation of the bandgap energy versus the Sb molar composition, *x* for the layer thickness T2SL *L*_T2SL_ = 4 nm, *L*_T2SL_ = 5 nm and *L*_T2SL_ = 6 nm.

Figure 5 shows the dependence of the T2SL bandgap energy on the Sb molar composition for T2SL with InAs/InAs_1−*x*_Sb*_x_* with the thicknesses *L*_T2SL_ = 4 nm, 5 nm and 6 nm for a strain-balanced and strained structure. The solid lines are marked bandgap energy SLs, whose thicknesses correspond to the balance condition after stresses, and the dashed line show the bandgap energy SLs that have a strain structure. It can be seen from the figures that these two types of structures provide a slightly different character of the change in the band gap as a function of the molar composition of the antimony. The change *x* has a greater effect on *E*_g_ in the strain samples.

**Figure 6:**
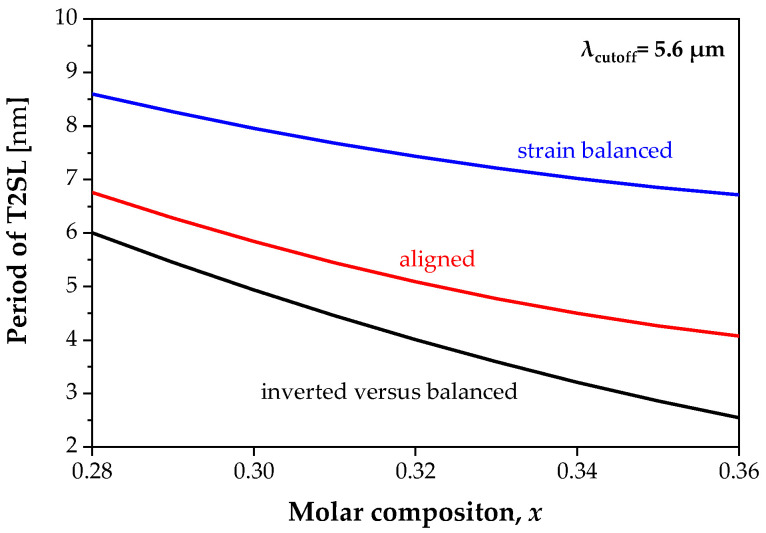
Theoretical simulation of the SL period versus molar composition *x* with the bandgap energy of 0.22 eV at *T* = 150 K.

The T2SL bandgap energy decreases with an increase in the size of the SL period. The introduction of additional strain lowers the *E*_g_ value, and this decrease is greater with the increase in the Sb molar composition in the InAs_1−*x*_Sb*_x_* layer. This is because the strain increases versus *x*. Figure 6 shows the dependence of the structure period with a Sb molar composition for a strain-balanced and strained structure, for T2SL 0.22 eV with a bandgap energy at *T* = 150 K corresponding to *λ*_cutoff_ = 5.6 µm. For structures with higher strains, the same bandgap energy can be reached with a much smaller SL period.

**Figure 7:**
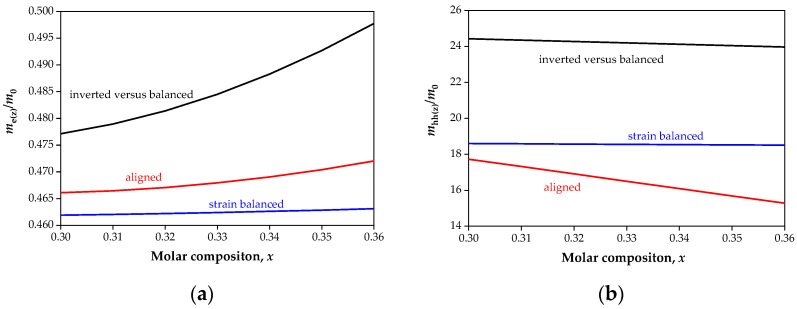
Theoretical simulation of the electrons’ (**a**) and holes’ (**b**) effective masses in the z-direction for the strain-balanced and strained SL versus of the Sb molar composition, *x* for the T2SL *L*_T2SL_ = 4 nm layer thickness at 150 K.

The electrons’ and holes’ effective masses were calculated of the dispersion curves as the second derivative of the bandgap energy by the wave vector ($\vec{k}$). For *k*_x_, the *k*_y_ directions of the Brillouin zone effective masses hardly change with the SL period and the molar composition *x*, but for direction *k*_z_, the dependence of the effective masses versus the SL period and *x* is significant. Figure 7 shows the theoretical simulation of the electrons’ (a) and holes’ (b) effective masses in the z-direction (*k*_z_) for the strain-balanced and strained T2SL versus the Sb molar composition for the *L*_T2SL_ = 4 nm period at 150 K.

It can be seen of Figure 7a,b that the electrons’ and holes’ effective masses for the balanced T2SL remain almost the same (blue curve) versus the Sb molar composition. For the strained T2SL with equal layer thicknesses (*L*_InAs_ = *L*_InAsSb_), the electrons’ effective masses increase slightly, and those of the holes decrease strongly. Analyzing the effective masses of the “inverted” T2SL, one can see that the mass of electrons increases very strongly versus the Sb molar composition, while the mass of heavy holes remains almost the same (it slightly decreases). From these analyses, it can be concluded that a strain-balanced structure is more stable than strained one.

**Figure 8:**
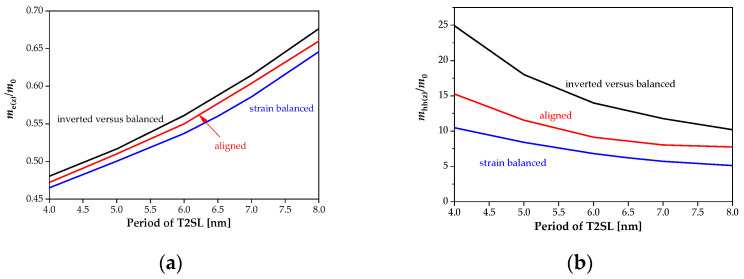
Theoretical simulation of the electrons’ (**a**) and holes’ (**b**) effective masses in the z direction for the strain-balanced and strained SL (the Sb molar composition for 0.22 eV T2SL bandgap energy).

The change in the SL period significantly affects the electrons’ and holes’ effective masses, especially in the *k*_z_ direction. Figure 8 shows the electrons’ (a) and holes’ (b) effective masses for direction *k*_z_ for the strain-balanced and strained SL with the Sb molar composition for the 0.22 eV SL bandgap energy. When the thickness of the *L*_T2SL_ increases, the holes’ effective masses decrease, while the electrons’ masses increase. The strains increase the electrons’ and holes’ masses for InAs/InAs_1−*x*_Sb*_x_* T2SL. For the balanced MWIR T2SLs InAs/InAs_1−*x*_Sb*_x_* with the period *L*_T2SL_ = 7 nm at *T* = 150 K, the calculated *e*_1_ and hh_1_ effective masses in the *k*_x_, *k*_y_ directions (parallel) and *k*_z_ directions (perpendicular) of the Brillouin zone are: *m*_e(x)_ = 0.019 *m*_0_, *m*_e(z)_ = 0.587 *m*_0_, *m*_hh(x)_ = 0.041 *m*_0_ and *m*_hh(z)_ = 5.677 *m*_0_. For example, in [9], the effective masses of the electrons have comparable values in the x directions and are on the level of 0.028 *m*_0_. Theoretical modelings by the authors [9] were conducted at the *T* = 100 K, which may allow the comparison of the order of magnitude and dynamics of change in electrons’ and holes’ effective masses. The obtained results show good agreement with the research conducted by the authors of the papers [8,9,20,21,22].

## 4. Conclusions

The theoretical assessment and analysis of the strain impact on InAs/InAs_1−*x*_Sb*_x_* T2SL bandgap energy and on the effective masses at 150 K were investigated. Three types of T2SL were compared: strain-balanced, aligned and inverted versus balanced. It was shown how to choose the T2SL period and the Sb molar composition of the InAs_1−*x*_Sb*_x_* to obtain devices for the MWIR range. The paper showed that the reduction in period T2SL and increase in InAs_1−*x*_Sb*_x_* antimony molar composition allows us to reach the same absorption edge. For structures with a higher strain, the same bandgap energy can be obtained with a much smaller T2SL period. As the strain increases, the absorption coefficient increases, and the carrier effective masses grow. We showed that the SL period significantly affects the electrons’ and holes’ effective masses, especially in the *k*_z_ direction. The InAs/InAs_1−*x*_Sb*_x_* T2SL energy band structure was calculated and the results show a good agreement with the results in the literature.

## Figures and Tables

**Table 1:** Parameters of the binary InAs and ternary InAs_0.70_Sb_0.30_ materials at *T* = 150 K.

Parameter	InAs	InAs_0.70_Sb_0.30_
*E*_g_ [meV]	391	180
Δ_SO_ [meV]	390	264
*m*_e_/*m*_0_	0.0244	0.0126
*γ*_1_	20.00	38.97
*γ*_2_	8.50	17.83
*γ*_3_	9.20	18.65

## Data Availability

Not applicable.
